# Cost Analysis of In-Home Telerehabilitation for Post-Knee Arthroplasty

**DOI:** 10.2196/jmir.3844

**Published:** 2015-03-31

**Authors:** Michel Tousignant, Hélène Moffet, Sylvie Nadeau, Chantal Mérette, Patrick Boissy, Hélène Corriveau, François Marquis, François Cabana, Pierre Ranger, Étienne L Belzile, Ronald Dimentberg

**Affiliations:** ^1^Research Centre on Aging, University Institute of Geriatrics of SherbrookeSherbrooke, QCCanada; ^2^Université de SherbrookeSherbrooke, QCCanada; ^3^Université LavalQuebec, QCCanada; ^4^Centre for Interdisciplinary Research in Rehabilitation and Social IntegrationQuebec, QCCanada; ^5^Université de MontréalMontreal, QCCanada; ^6^Centre for Interdisciplinary Research in Rehabilitation of Greater MontrealMontreal, QCCanada; ^7^Centre de recherche de l'Institut universitaire en santé mentale de QuébecQuebec, QCCanada; ^8^Centre hospitalier universitaire de QuébecQuebec, QCCanada; ^9^Centre hospitalier universitaire de SherbrookeSherbrooke, QCCanada; ^10^Hôpital Jean-TalonMontreal, QCCanada; ^11^McGill UniversityMontreal, QCCanada; ^12^St Mary's HospitalMontreal, QCCanada

**Keywords:** telemedicine, economics, cost analysis, knee arthroplasty

## Abstract

**Background:**

Rehabilitation provided through home visits is part of the continuum of care after discharge from hospital following total knee arthroplasty (TKA). As demands for rehabilitation at home are growing and becoming more difficult to meet, in-home telerehabilitation has been proposed as an alternate service delivery method. However, there is a need for robust data concerning both the effectiveness and the cost of dispensing in-home telerehabilitation.

**Objective:**

The objective of this study was to document, analyze, and compare real costs of two service delivery methods: in-home telerehabilitation and conventional home visits.

**Methods:**

The economic analysis was conducted as part of a multicenter randomized controlled trial (RCT) on telerehabilitation for TKA, and involved data from 197 patients, post-TKA. Twice a week for 8 weeks, participants received supervised physiotherapy via two delivery methods, depending on their study group allocation: in-home telerehabilitation (TELE) and home-visit rehabilitation (VISIT). Patients were recruited from eight hospitals in the province of Quebec, Canada. The TELE group intervention was delivered by videoconferencing over high-speed Internet. The VISIT group received the same intervention at home. Costs related to the delivery of the two services (TELE and VISIT) were calculated. Student’s *t* tests were used to compare costs per treatment between the two groups. To take distance into account, the two treatment groups were compared within distance strata using two-way analyses of variance (ANOVAs).

**Results:**

The mean cost of a single session was Can $93.08 for the VISIT group (SD $35.70) and $80.99 for the TELE group (SD $26.60). When comparing both groups, real total cost analysis showed a cost differential in favor of the TELE group (TELE minus VISIT: -$263, 95% CI -$382 to -$143). However, when the patient’s home was located less than 30 km round-trip from the health care center, the difference in costs between TELE and VISIT treatments was not significant (*P*=.25, .26, and .11 for the <10, 10-19, and 20-29 km strata, respectively). The cost of TELE treatments was lower than VISIT treatments when the distance was 30 km or more (30-49 km: $81<$103, *P*=.002; ≥50 km: $90<$152, *P*<.001).

**Conclusions:**

To our knowledge, this is the first study of the actual costs of in-home telerehabilitation covering all subcosts of telerehabilitation and distance between the health care center and the patient’s home. The cost for a single session of in-home telerehabilitation compared to conventional home-visit rehabilitation was lower or about the same, depending on the distance between the patient’s home and health care center. Under the controlled conditions of an RCT, a favorable cost differential was observed when the patient was more than 30 km from the provider. Stakeholders and program planners can use these data to guide decisions regarding introducing telerehabilitation as a new service in their clinic.

**Trial Registration:**

International Standard Registered Clinical Study Number (ISRCTN): 66285945; http://www.isrctn.com/ISRCTN66285945 (Archived by WebCite at http://www.webcitation.org/6WlT2nuX4).

## Introduction

The rapid growth in the number of total knee arthroplasty (TKA) surgeries per year and shorter hospital stays have greatly increased demand for outpatient rehabilitation services for patients returning home more quickly after hospital discharge. However, this increasing need has not been followed by an increase in accessibility to rehabilitation services after TKA, in-home care, or outpatient clinics. As part of a worldwide situation [1], in the province of Quebec, Canada, it has been shown that actual rehabilitation services cannot meet needs, both because the services are not accessible everywhere and because of a lack of health care professionals [2-4]. To increase accessibility to rehabilitation, alternative approaches to delivering these services must be considered [5].

Teletreatment, which is defined as care and treatment delivered remotely between patients and health care professionals with the support of communication and information technology [6], is a recent approach that can be relevant to rehabilitation patients. As opposed to teleconsultation, teletreatment has an essential characteristic, which is the repeated delivery of synchronous services through videoconferencing to provide real-time interactions between patients and clinicians. Another characteristic is that teletreatment may be delivered between two health care centers or between a health care center and the patient’s home under direct supervision (ie, in-home teletreatment). In this paper, we focus on in-home teletreatment.

Our main interest is to increase access to, and efficiency of, rehabilitation services after TKA through the use of teletreatment between the health care center and patient’s home. In this context, the feasibility of providing in-home telerehabilitation following joint replacement surgery has been studied previously [7-8]. Those pilot projects showed the feasibility of telerehabilitation for TKA. However, the small sample size or lack of a control group limited the generalizability of those results. No real cost analysis was included in those studies. Only a theoretical estimate of the costs associated with teletreatment compared to home visits suggested that teletreatment had the potential to be less costly than home visits [7]. However, that theoretical estimate cannot be generalized and a rigorous cost analysis is needed to provide robust data on the cost of teletreatment. In a recent literature review [9], 35 articles were found, where the majority were about cost-effectiveness of telemedicine in general. Some, but not all, studies demonstrate that telemedicine can reduce the costs. Up to now, no cost analysis on teletreatment between the health care center and the patient’s home using an audiovideo synchronous system has been done.

This cost analysis was embedded in the telerehabilitation for knee arthroplasty (TelAge) randomized controlled trial (RCT) on the effectiveness of in-home telerehabilitation. The TelAge project is a multicenter randomized trial, involving three research centers located in Quebec City, Sherbrooke, and Montreal, and well as eight hospitals [10]. Thus, the purpose of this paper was to compare costs related to in-home teletreatment versus conventional home-visit rehabilitation following TKA.

## Methods

### TelAge Randomized Controlled Trial Design

The project was approved by ethics committees of each hospital and the participants’ written consent was obtained prior to their inclusion. The RCT was registered with the International Standard Registered Clinical Study Number (ISRCTN) registry (ISRCTN66285945).

Participants were recruited from the surgical waiting lists of orthopedic surgeons in each hospital. To be included in the study, they had to meet the following inclusion criteria: (1) were waiting for a primary TKA after a diagnosis of osteoarthritis, (2) returning back home after hospital discharge, (3) living in an area served by high-speed Internet services (at least 512 kbps in upload), and (4) living within a 1-hour driving distance from the treating hospital. Patients were excluded if they met the following criteria: (1) had health conditions that could interfere with tests or the rehabilitation program, including lower limb surgery in the last 9 months, (2) were planning a second lower limb surgery within 4 months, (3) had cognitive or collaboration problems, (4) had major postoperative complications, or (5) had weight-bearing restrictions for a period longer than 2 weeks after surgery.

**Figure 1:**
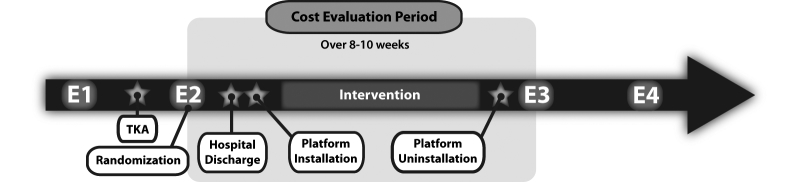
TelAge clinical randomized controlled trial study design including cost evaluation period.

After their surgery, patients were block randomized by random generator with sealed envelopes into either the in-home telerehabilitation (TELE) experimental group or the home-visit (VISIT) control group. For both groups, the goal of the physiotherapy program was to recover function—only the delivery method differed. The duration and frequency of the supervised physiotherapy sessions were standardized for both groups and consisted of two 45-minute sessions per week for 8 weeks. Patients were assessed four times by a blind assessor—twice before and twice after the intervention—to evaluate rehabilitation efficacy, along the following timeline: (1) at baseline (ie, 1 month or less before TKA) (E1), (2) 1 to 2 days before discharge from hospital (E2), (3) 2 months postdischarge (E3), and (4) 4 months postdischarge (E4). Figure 1 shows the study design, including the cost evaluation period. Results obtained for efficacy demonstrated the noninferiority of in-home telerehabilitation and support its use as an effective alternative to face-to-face service delivery for TKA patients after hospital discharge [11].

**Figure 2:**
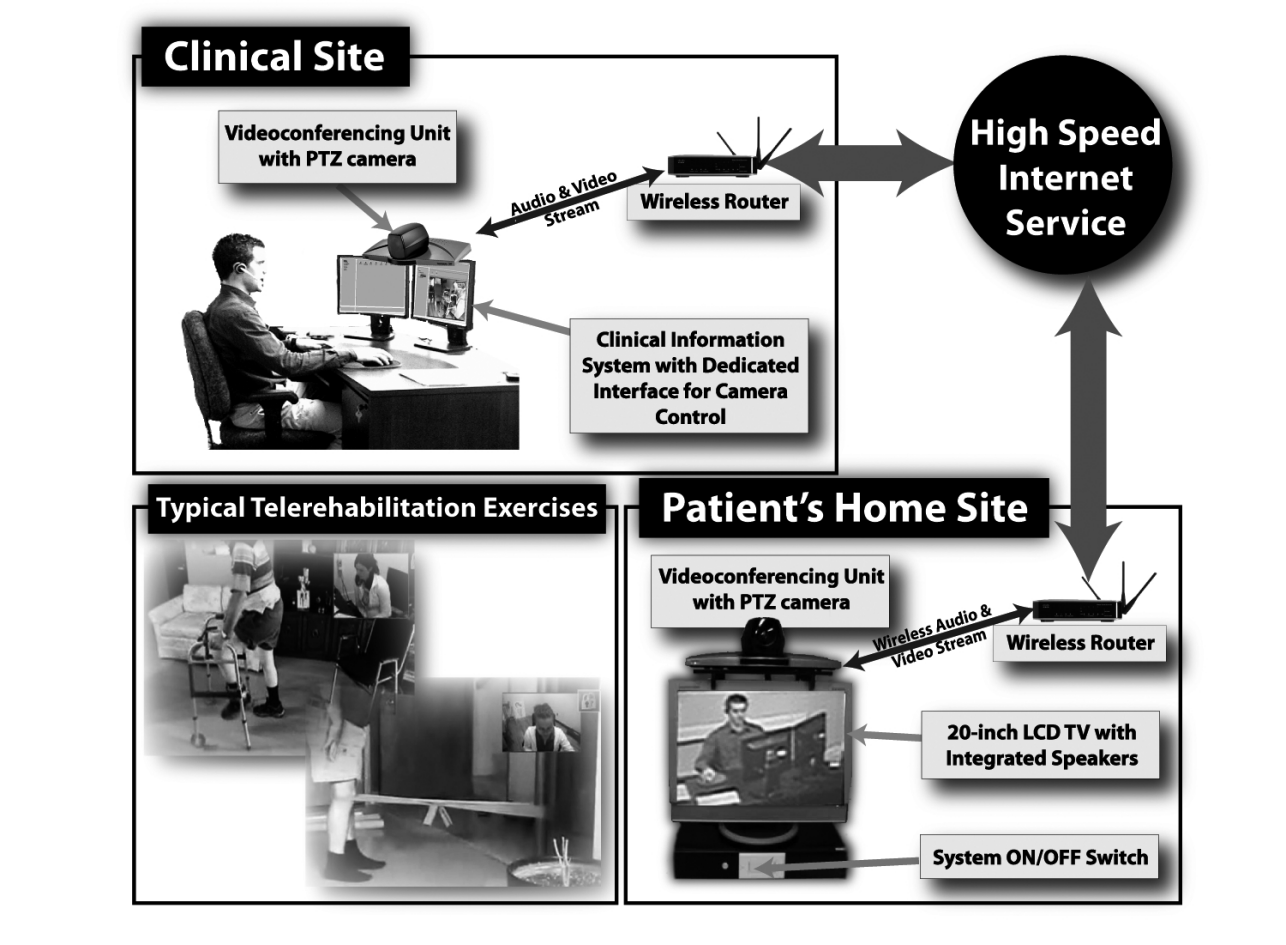
Telerehabilitation platform.

The telerehabilitation platform included various components to provide a user-friendly videoconferencing experience for both the clinician and the patient at home. The core was the videoconferencing system (Tandberg 550 MXP), which used an H.264 video codec and incorporated a pan-tilt-zoom (PTZ) camera with wide-angle lens and omnidirectional microphone. On the patient side, the system was mounted over a 20-inch LCD screen, which displayed the video received from the other end. Audio was played using external speakers placed on both sides of the screen, as the internal LCD screen speakers are rarely sufficient to provide a satisfactory experience. Video and audio data were encrypted and transmitted over a high-speed Internet connection—at the time of the TelAge RCT, minimum bandwidth for both upload and download was 512 kbps. On the clinician side, a computer with dedicated software (TeRA) was added to the videoconference link to enable user-friendly control and monitoring of videoconferencing sessions, near- and far-end camera controls, built-in clinical tests, photo and video recordings, and acquisition and display of external sensors and peripherals (Figure 2).

### Cost Measurement

Costs were measured during the intervention period—between E2 and E3—for both groups. All cost-related data were directly collected by the following: (1) physiotherapists after each session (ie, intervention, travel, technical problems), and (2) technicians after each installation/uninstallation (ie, travel, technical problems). Cost-related data were collected using a standardized cost sheet during the period from hospital discharge to 2 months postdischarge (ie, the end of rehabilitation intervention). Other costs, such as Internet service and technical/clinical equipment amortization, were calculated using the real cost of the service and purchase price of the equipment, respectively, at the time of the TelAge RCT.

This economic evaluation followed international guidelines for conducting a cost analysis alongside a clinical RCT [12]. The economic analysis was based on a health sector perspective, meaning that only costs relevant to the health center were considered, and not costs relevant to the patient. In addition, only costs related to the delivery of the two services—TELE and VISIT—were taken into account [12]. Costs were divided into two categories: (1) costs related to the clinical aspects, and (2) costs related to the technology. For each of these cost categories, direct costs were defined as essential for the delivery of the clinical intervention, and indirect costs as being related to the intervention without being part of it.

More precisely, direct clinical time included duration of the interaction between physiotherapist and participant to deliver the intervention in both groups. Indirect clinical time was defined as the following: (1) time without direct contact with the participant, but which was required to optimize the intervention (ie, scheduling, file preparation, planning the treatment, writing the report and follow-up notes, phone follow-up with orthopedic surgeon), (2) travel time and kilometer (km) allocation for use of the physiotherapist’s car—only for the VISIT group, and (3) clinical equipment amortization, which is the theoretical time duration of use of the technology. Round-trip distance from the health care center (ie, hospital where the research center involved in the project was located) to the patient’s home was calculated for each patient in both groups.

Direct technology costs—only for the TELE group—are the costs in Canadian dollars for acquiring clinician and patient telerehabilitation platform kits as illustrated in Figure 2. The cost of the equipment was amortized assuming 3 years of use at the rate of 17 hours per week (ie, about 50% of a 35-hour week). Indirect technology costs included the following: (1) time spent on transportation and installation and uninstallation of the platform at home (ie, technician’s salary and kilometer allocation), (2) the cost to provide a new connection or upgrade high-speed Internet from the Internet service providers for the duration of the intervention (ie, connection fee plus 2 months of Internet service), and (3) technician’s time and travel to deal with technical problems before/during/after the physiotherapists’ sessions.

The standard hourly salary in effect in the public health system in Quebec at the time of the TelAge RCT was applied to the time spent by the physiotherapists and technicians—Can $45.22/hour and Can $22.09/hour, respectively. Travel expenses were calculated as distance in kilometers multiplied by Can $0.40. In addition, to determine the cost of the whole treatment (ie, 16 sessions), decision makers may be interested in the net costs of giving one TELE or one VISIT treatment, for instance, the cost per treatment. For that analysis, only costs of treatments actually received were taken into account. Moreover, the distribution of costs in each delivery-service method was analyzed. Table 1 shows the breakdown of clinical costs for both study groups.

**Table 1:** Breakdown of costs per treatment for telerehabilitation and home visits.

Cost category		Description	Duration	Professional cost^a^calculation	
				TELE	VISIT
**Clinical**					
	**Direct time**				
		Contact with the participant	Hours	Hours x $45.22^b^	Hours x $45.22
	**Indirect time**				
		Planning the session	Hours	Hours x $45.22	Hours x $45.22
		Follow-up with orthopedic surgeon	Hours	Hours x $45.22	Hours x $45.22
		Writing the report and follow-up notes	Hours	Hours x $45.22	Hours x $45.22
		Travel	Hours, km	N/A^c^	Hours x $45.22, km x $0.40
		Clinical equipment amortization	16 treatments	$0.63 per treatment ($10/16 treatments)	$0.63 per treatment ($10/16 treatments)
**Technology**					
	Installation/uninstallation of technology			Travel time (hours) x $22.16^d^, km x $0.40	N/A
	Internet costs (high speed)^e^			$7.63 per intervention	N/A
	Technical equipment amortization^f^			$4.88 per intervention	N/A
**Technical problems**					
	Technician			Hours x $22.16	N/A
	Physiotherapist			Hours x $45.22	N/A

^a^All costs are in Canadian dollars.

^b^Physiotherapist mean hourly salary in the public system in Quebec.

^c^Not applicable (N/A).

^d^The hourly rate at the higher echelon of the salary scale of a technician.

^e^Cost for 2 months (16 sessions): $122.08/16 sessions = $7.63 per session.

^f^Cost for 1 clinician kit ($5760) and 1 patient kit ($7200) amortized over 3 years based on 50% of usage per week (17 hours): $5760 + $7200 = $12,960. Hours telerehabilitation: 3 years x 52 weeks/year x 17 hours (50% usage)/week = 2652 hours. Cost per treatment: $12,960/2652 hours = $4.88.

### Sensitivity Analysis

As time spent on travel is an important cost factor for the VISIT group, the distance between the health care center and patient's home is an important variable that must be taken into account when comparing costs between VISIT and TELE treatments. To consider the impact of this variable, a sensitivity analysis was performed on the basis of the distance between the patient’s home and health care center for the physiotherapist delivering the treatment (VISIT) or the technician installing/uninstalling the technology (TELE). Another way to look at this sensitivity analysis is to consider that time spent on travel may differ in metropolitan, urban, and rural areas, for instance, travelling 5 km in a metropolitan area can take more time than in urban and rural areas. Knowing that, the analyses were done separately for each site.

### Statistical Analyses

All analyses were performed using SAS/STAT software, version 9.3 of the SAS System for Windows (SAS Institute Inc, Cary, NC, USA). First, the two groups of patients were compared using sociodemographic characteristics. For the continuous variables, Student’s *t* tests were conducted using the TTEST procedure. For the categorical variables, chi-square tests were computed using the FREQ procedure. Then the treatment costs were compared between the TELE and VISIT groups by Student’s *t* tests using the TTEST procedure. Finally, considering the fact that travel time is an important cost factor for the VISIT group, the round-trip distance from the health care center to the patient’s home was calculated for each patient in both groups. Patients were then grouped into five distance strata: <10, 10-19, 20-29, 30-49, ≥50 km. In addition, analyses were conducted per site (ie, Montreal, Quebec City, and Sherbrooke) to take into account metropolitan, urban, and rural areas. Two-way analyses of variance (ANOVAs) with interaction were conducted to compare costs per treatment between the two groups and between distance strata in the total sample and for each site. When the interaction term was significant, post hoc comparisons were examined to compare the two groups within each distance stratum. When appropriate, distance strata were grouped to simplify interpretation of the results. SAS’s general linear model (GLM) procedure was used with SLICE option in the least squares means (LSMEANS) statement to obtain the post hoc comparisons.

## Results

### Participants

A total of 258 patients needing TKA were recruited for the TelAge RCT [10]. Postsurgery, 53 (20.5%) participants were excluded and the 205 (79.5%) who remained were block randomized to the TELE group (104/205, 50.7%) or VISIT group (101/205, 49.3%). Of the 104 TELE group participants, 6 (5.8%) were excluded before the first follow-up assessment (E3) because they were unsatisfied with randomization (3), had poor Internet connection (2), or had self-perception of complete recovery (1). In the TELE group, only 1 of the participants was excluded following the randomization because no physical therapist was available. Moreover, 1 participant in the TELE group was excluded from the analysis because he did not attend any of the rehabilitation sessions. The cost analysis was thus performed with 97 and 100 participants in the TELE and VISIT groups, respectively. The sociodemographic characteristics of the final sample used in the cost analysis and the participants’ locations expressed as distance to the treatment center are presented in Table 2.

The characteristics of the participants were comparable in each group for every sociodemographic variable measured, except for participants living alone (*P*=.04), where about twice as many participants in the TELE group lived alone as in the VISIT group. Also, participants in the TELE group lived farther away from the health care center than those in the VISIT group—mean round-trip distance for the TELE group was 59 km (SD 67) and for the VISIT group was 34 km (SD 35) (*P*=.002).

**Table 2:** Descriptive statistics at baseline of the sample included in the cost analysis (n=197).

Sociodemographic characteristic	TELE (n=97), mean (SD) or n (%)	VISIT (n=100), mean (SD) or n (%)	*P* value^a^
Age (years), mean (SD)	65 (8)	67 (8)	.11
Body mass index (kg/m^2^), mean (SD)	34.6 (7.6)	33.1 (5.6)	.12
Comorbidity Index (%), mean (SD)	23 (12)	21 (9)	.16
Functional ability before TKA (WOMAC^b^ in %), mean (SD)	53 (19)	54 (17)	.74
Round-trip distance from health care center to patient’s home (km), mean (SD)	59 (67)	34 (35)	.002
Sex (males), n (%)	40 (41)	55 (55.0)	.05
Operated knee (right), n (%)	45 (46)	51 (51.0)	.52
Previous lower limb surgery, n (%)	44 (45)	49 (49.0)	.66
Living alone, n (%)	20 (21)	10 (10.0)	.04

^a^ *P* values are from Student’s *t* tests for continuous variables and from chi-square tests for categorical variables.

^b^Western Ontario and McMaster Universities Arthritis Index (WOMAC).

### Costs Related to Treatment Delivery (Treatments Received and Cancelled)

In the randomized trial, some participants from the TELE group and VISIT group did not receive all 16 scheduled treatments due to medical, technical, or personal reasons. Average treatment frequency was 16.0 (SD 0.2) for the VISIT group and 15.3 (SD 2.1) for the TELE group (*P*=.004). Because there are expenses associated with cancelled treatments (ie, travel to the home without giving the treatment, technician time to solve technological problems), the cost analysis took into account real costs, for instance, the cost of treatment given plus cost of cancelled treatment (Table 3). Real cost analysis shows a cost differential in favor of the TELE group (TELE minus VISIT: Can -$263, 95% CI -$382 to -$143), meaning that for each participant’s total intervention, telerehabilitation saves the health care system 18% of the costs incurred for conventional rehabilitation following TKA.

**Table 3:** Costs of telerehabilitation and home visits.

Cost	TELE ($) (n=97), mean (SD)	VISIT ($) (n=100), mean (SD)	TELE-VISIT ($), difference (95% CI)	(TELE-VISIT)/VISIT (%), difference/VISIT (95% CI)	*P* value
Total cost^a^	1224 (241)	1487 (553)	-263 (-382 to -143)	-18 (-26 to -10)	<.001
Cost per treatment^b^	80.99 (26.60)	93.08 (35.70)	-12.09 (-20.91 to -3.27)	-13 (-23 to -4)	.008

^a^Total cost is for the total intervention, which includes all received and cancelled treatments.

^b^Cost per treatment is for one treatment and is based only on treatments received.

### Cost per Treatment

Cost per treatment shows that the mean cost of treatment for the VISIT group was Can $93.08 (SD $35.70) versus Can $80.99 (SD $26.60) for TELE group. The differential cost between the two groups was 13% in favor of the TELE group (TELE minus VISIT: Can -$12.09, 95% CI -$20.91 to -$3.27) (Table 3).

### Total Cost Distribution for Each Group

Table 4 outlines the distribution related to direct and indirect categories of costs. As expected, total direct clinical costs—TELE $36.59, VISIT $41.65—were quite similar. However, even though rehabilitation session length was standardized in the protocol, treatment duration was longer in the VISIT group—TELE 47.6 (SD 10.2) minutes, VISIT 54.4 (SD 12.1) minutes (*P*<.001). Indirect costs for the VISIT group were higher than for the TELE group because of the costs associated with travel to the patient’s home—TELE $11.51, VISIT $51.43. However, technology costs for the TELE group totaled $32.88, which to some extent counterbalanced the travel costs for the VISIT group.

As expected, the travel cost for the VISIT group is an important cost-driven variable—49% of the total cost. Interestingly, costs related to technology support during teletreatment, outside of the expected time for equipment installation/uninstallation, did not significantly affect the cost—1.5% of total cost.

At a micro level, it is somewhat surprising to see discrepancies between some subcosts. For example, therapists spent the same proportion of time with the patient in both groups—TELE 44%, VISIT 44%—but a larger proportion of time was spent in the TELE group preparing for the treatment—TELE 5%, VISIT 2%—and on follow-up—TELE 9%, VISIT 4%.

### Sensitivity Analysis

There was a difference in costs per treatment between the two groups, and this difference was not the same in the five distance strata (*P*<.001 for interaction) (Table 5). When the patient’s home was less than 30 km round-trip from the health care center, the difference in costs between TELE and VISIT treatments was not significant (*P*=.25, .26, and .11 for the <10, 10-19, and 20-29 km strata, respectively). However, TELE treatments were less costly than VISIT treatments when the distance was 30 km or more (30-49 km, Can $81<$103, *P*=.002; ≥50 km, $90<$152, *P*<.001).

For urban and rural areas, TELE treatments were less expensive than VISIT treatments when the round-trip distance was 50 km or more (Can $97<$144, *P*<.001) (Table 6). For metropolitan areas, TELE treatments were cheaper than VISIT treatments when the round-trip distance was 30 km or more (Montreal, Can $80<$152, *P*<.001; Quebec City, $82<$108, *P*=.001).

**Table 4:** Breakdown of costs per treatment (telerehabilitation and home visits).

Cost category			TELE		VISIT	
			Can $	%	Can $	%
**Clinical**						
	**Direct**					
		Treatment	35.85	44	41.02	44
		Clinical equipment amortization^a^	0.74	1	0.63	1
		Total	36.59	45	41.65	45
	**Indirect**					
		Time related to preparation for the treatment	4.48	5	2.26	2
		Follow-up with patients	7.03	9	3.55	4
		Travel to participant’s home (time + km allocation)	N/A^b^	N/A	45.62	49
		Total	11.51	14	51.43	55
	Total clinical costs		48.10	59	93.08	100
**Technological**						
	**Direct**					
		Technical equipment amortization^a^	5.81	7.2	N/A	N/A
		Installation/uninstallation of technology	18.12	22.4	N/A	N/A
		Internet costs	7.63	9.4	N/A	N/A
		Technical problem resolution time and travel time (technician)	1.25	1.5	N/A	N/A
		Technical problems after sessions by the physiotherapist	0.08	0.1	N/A	N/A
	Total technological costs		32.88	41	N/A	N/A
**Total costs**			80.99	100	93.08	100

^a^The clinical and technical equipment amortization costs are slightly different from those in Table 1 for the TELE group because some patients did not receive all 16 scheduled treatments.

^b^Not applicable (N/A).

**Table 5:** Cost sensitivity analysis based on round-trip distance from health care center to patient’s home in the total sample (n=197).

Distance (km)	Costs per treatment (Can $)				*P* value^a^
	TELE		VISIT		
	n (%)	Mean (SD)	n (%)	Mean (SD)	
<10	12 (6.1)	71.0 (9.8)	21 (10.7)	63.9 (7.4)	.248
10-19	24 (12.2)	69.5 (8.5)	27 (13.7)	74.8 (10.5)	.260
20-29	14 (7.1)	73.6 (10.1)	19 (9.6)	83.1 (8.3)	.112
30-49	12 (6.1)	81.3 (13.1)	13 (6.6)	102.7 (19.5)	.002
≥50	35 (17.8)	90.2 (24.4)	20 (10.2)	151.6 (30.6)	<.001

^a^Two-way ANOVA with interaction was conducted to compare costs per treatment between the two groups and between the five distance strata (*P*<.001 for interaction). *P* values shown in the table come from post hoc comparisons.

**Table 6:** Cost sensitivity analysis based on round-trip distance from health care center to patient’s home, calculated separately for each site.

Site			Costs per treatment (Can $)				Interaction *P* value^a^	Post hoc *P* value
			TELE		VISIT			
		Distance (km)	n (%)	Mean (SD)	n (%)	Mean (SD)		
**Urban and rural area**								
	**Sherbrooke (n=109)**							
		< 50	34 (31.2)	69.8 (8.3)	46 (42.2)	71.4 (10.4)	<.001	.684
		≥ 50	20 (18.3)	97.3 (28.8)	9 (8.3)	143.5 (26.1)		<.001
**Metropolitan areas**								
	**Montreal** **(n=37)**							
		< 30	4 (11)	74.6 (10.4)	8 (22)	92.4 (12.3)	.002	.192
		≥ 30	13 (35)	80.2 (9.0)	12 (32)	152.0 (34.9)		<.001
	**Quebec City** **(n=50)**							
		< 30	14 (28)	73.7 (10.6)	16 (32)	75.0 (9.0)	.001	.815
		≥ 30	11 (22)	81.7 (15.7)	9 (18)	107.7 (28.6)		.001

^a^For each site, a two-way ANOVA with interaction was conducted to compare costs per treatment between the two groups and between the distance strata. Results shown in the table are *P* values for interaction between groups and distance strata, and *P* values from post hoc comparisons.

## Discussion

### Principal Findings

The purpose of this study was to document costs for in-home teletreatment versus conventional home-visit rehabilitation following TKA based on an RCT. To our knowledge, this is the first cost analysis of in-home telerehabilitation involving an assessment of the cost structure for this type of service delivery versus traditional in-home, face-to-face rehabilitation. One unique facet of our study is the inclusion of all subcosts related to telerehabilitation (ie, equipment amortization, installation/uninstallation, technical problems related to teletreatment). Our results show that delivering physiotherapy by telerehabilitation post-TKA is less expensive than providing the same service in home visits if the distance between the health care center and patient’s home is more than 30 km. Overall, when we take into account all direct and indirect costs of the two approaches, teletreatment between the health care center and patient’s home is less costly than home visits by at least 18%.

In the context of cost constraints in the health care system, is the cost savings of 18% sufficient to introduce in-home telerehabilitation? This result did not specifically take into account one major cost-driven factor related to home visits, which is proximity of the participant’s home to the health care center (ie, sensitivity analyses). The inherent hypothesis that comes to mind is that proximity of health care centers to patients’ homes can significantly influence the cost of the VISIT group—the farther away a participant lives, the higher the costs related to home-visit rehabilitation. For example, in the VISIT group, 16 round-trips were needed to provide treatment in the home by the physiotherapist, but only two round-trips were needed for the TELE group (ie, installation/uninstallation) by the technician. To take into consideration the proximity of the participant’s home to the health care center, a sensitivity analysis clearly showed that if travel distance is less than 30 km, round-trip, differential costs between the two alternatives decline. On the other hand, if round-trip travel distance is longer than 30 km, the cost differential increases dramatically, reaching 123% for 50 km or more. This is an important result for those who want to implement telerehabilitation—to benefit from cost savings, telerehabilitation must involve patients who live at least a 30 km drive, round-trip, from the clinic if the resources are available. In addition, it would be more efficient to use specialized health care providers to administer treatments than to spend their time travelling, as was done in the VISIT group.

Moreover, if we consider traffic in metropolitan, urban, and rural areas as a confounding variable to proximity of the participant’s home to the health care center, we observed the same tendency, with the difference being that in the urban and rural area (Sherbrooke), a difference of 50 km was needed to keep the cost savings of the TELE versus the VISIT group. This study shows that costs were not higher for the home-visit group, which could be of importance to stakeholders and managers.

As a novel piece of the puzzle to understand costs of telerehabilitation compared to home visits, examination of the subcosts of the two approaches produced some interesting results. For that analysis, only costs of the treatment actually delivered were considered. Direct treatment time was not the same in both groups—it was less in the TELE group. Even though there was a standardization of the treatment in both groups in the research protocol, this difference can be attributed to taking less time to discuss with the patient or less time between exercises because of the virtual mode of communication. However, we saw that the proportion of time spent on preparing for the treatment and following up after therapy was more than double in the TELE group (Table 4). We attribute this result to a virtual session inducing more caution on the part of the therapist. Being in front of the screen waiting for the time scheduled with the patient may lead to taking more time to prepare for the session as opposed to driving a car to go to the patient’s home. Further research should assess these aspects in a qualitative study embedded in another trial.

As expected, travel drove the costs of home visits (ie, 49% of total costs). The technology of the TELE group was also costly (ie, 41% of total), but not enough to counterbalance travel costs. Looking at the subcategories of the 41% technology costs, 7.2% of the costs were attributable to equipment amortization and 9.4% to telecommunication (ie, high-speed Internet). At the time of the TelAge RCT (2008-2013), the videoconferencing equipment purchase costs and telecommunication costs were much higher than for comparable equipment and services today. Comparable videoconferencing equipment is available for less than half the purchase price used in our analyses. Telecommunication costs for activation fees and bandwidth are significantly lower as accessibility and use of high-speed Internet have now reached unprecedented levels, even with older adults, and bandwidth costs have declined. As the cost per treatment for the technological factors of in-home telerehabilitation was only 16% of the total costs, this would not appear to be a determining variable in our results. However, the exponential evolution of these technological factors in today’s technological context (ie, lower cost of videoconferencing, ubiquity, and performance of telecommunication networks) could impact the cost differential between the TELE group and the face-to-face VISIT group services delivery by lowering the barrier to entry of the TELE option when scaling up in-home visit rehabilitation.

Regarding internal validity, information bias was minimized by using specific procedures. First, data collection was standardized with cost forms and information was collected immediately after each intervention (ie, physiotherapy session). Second, the standardized cost forms were pretested. This assured us of the specific details required to calculate all subcosts. For each subcost, the person involved in service delivery (ie, technician, engineer, physiotherapist, study coordinator, etc) completed the appropriate form the same day that the intervention occurred. Following this rigorous process, memory bias should have been minimized. Finally, to decrease missing data, the site coordinator checked the data for completeness. With all these precautions, we are confident in the validity of the cost data.

Selection bias was controlled by the randomization procedure in the TelAge RCT leading to two equivalent groups regarding sociodemographic variables [10]. The only exception was the mean distance per round-trip between the health care center and the patient’s home, which was greater in the TELE group. If this discrepancy had an effect, it should have tended to increase the 18% cost difference observed in the TELE group.

### Clinical Implications of This Study

We already know that teletreatment for TKA is efficient [10]. The results of the TelAge clinical trial demonstrated that the TELE group treatment was at least as clinically effective as home visits. That was a key issue in order to justify implementation of telerehabilitation. The cost analysis conducted in this trial supports the use of teletreatment in cost-saving conditions. If teletreatments are given the same way as in this study, cost savings can range from 18% to 123%, depending on travel time from the health care center to the patient’s home. The cost savings of at least 18% is a new way to increase budget for rehabilitation services. For example, 18% of a $1 million budget means cost-savings of $180,000. That means that at least two more health professionals can be hired over the next year. Moreover, with sensitivity analysis, this increase may be more important for some facilities. With well-known budget constraints being experienced in health care, worldwide, we must investigate innovative ways of managing and stretching budgets. We believe that health care centers could use teletreatment for a subpopulation of their patients. We think that the hypothesis that 50% of the scheduled treatment time can be dedicated to telerehabilitation—because not all patients can/want/must receive teletreatment in a general hospital—is conservative and reliable. We need to understand that telerehabilitation is a compromise for the therapist—he/she may not want to treat all his/her patients in a virtual way, perhaps only those who cannot reach outpatient clinics or those for whom home care is not feasible.

### Conclusions

In the context of increasing access to rehabilitation, our study demonstrated the cost savings of teletreatment compared to home visits for patients post-TKA. Managers can now decide whether to implement telerehabilitation based on robust clinical and economic data. Given the recent attention that telerehabilitation is drawing on an international level, it would be interesting in future research to carry out a large-scale study about one of the major practical issues related to the provision of telemedicine services to a specific patient population.

## Abbreviations

ANOVA: analysis of variance
CIHR: Canadian Institutes of Health Research
E1: assessment time, at baseline
E2: assessment time, 1 to 2 days before discharge from hospital
E3: assessment time, 2 months postdischarge
E4: assessment time, 4 months postdischarge
GLM: general linear model
ISRCTN: International Standard Registered Clinical Study Number
km: kilometer
LSMEANS: least squares means
N/A: not applicable
PTZ: pan-tilt-zoom
RCT: randomized controlled trial
TelAge: telerehabilitation for knee arthroplasty
TELE: in-home telerehabilitation (experimental) treatment
TKA: total knee arthroplasty
VISIT: home-visit (control) treatment
WOMAC: Western Ontario and McMaster Universities Arthritis Index
